# Endobronchial carcinoid presenting as Focal bronchiectasis in a young woman with systemic lupus erythematosus

**DOI:** 10.1002/ccr3.4840

**Published:** 2021-10-28

**Authors:** Noreen Nasir, Safia Akhlaq, Talha Shehzad, Saulat Fatimi

**Affiliations:** ^1^ Section of Internal Medicine Department of Medicine Aga Khan University Karachi Pakistan; ^2^ Section of Pulmonology Department of Medicine Aga Khan University Karachi Pakistan; ^3^ Section of Cardiothoracic Surgery Department of Surgery Aga Khan University Karachi Pakistan

**Keywords:** endobronchial carcinoid tumor, malignancy in SLE, Systemic Lupus Erythematosus

## Abstract

Non‐resolving pulmonary symptoms in a patient with SLE require evaluation to exclude rare pulmonary lesions, such as carcinoid tumors.

## Graphical abstract

This case affirms the fact that non‐resolving pulmonary symptoms in a patient with SLE require evaluation to exclude rare pulmonary lesions, such as carcinoid tumors.

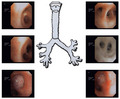

## 1 INTRODUCTION

Although the literature review suggests an increase of lung malignancy in Systemic Lupus Erythematosus (SLE), it is unusual to encounter endobronchial carcinoid tumors in a patient with systemic lupus. We highlight a case of a young lady with SLE who presented with worsening shortness of breath with one‐month history of high‐grade fever and productive cough. After a series of initial investigations, a bronchoscopic biopsy showed an endobronchial carcinoid tumor obstructing left upper lobe bronchus, resulting in a focal bronchiectasis and recurrent infective exacerbation.

Bronchial carcinoids are rare neuroendocrine tumors, accounting for <5% of all bronchopulmonary tumors.^1^ According to the WHO, there are four histological types of pulmonary neuroendocrine tumors:^2^ These include typical carcinoids,atypical carcinomas, small cell carcinomas and large‐cell neuroendocrine carcinomas.^2^ The most common primary endobronchial lung neoplasm is the "Typical carcinoid" accounting for 80‐90% of pulmonary carcinoid tumors.^3^ The definitive management of carcinoid tumors is surgical resection which is the mainstay of treatment. ^4^ Bronchial carcinoids can present with an atypical presentation, and awareness among clinicians is essential for prompt diagnosis and management.^5^ Systemic lupus erythmatosus (SLE) is a complex autoimmune connective tissue disease.^6^ Although, literature review suggests an increased risk of lung malignancy in SLE, it is exceedingly rare to encounter endobronchial carcinoid tumor in a patient with systemic lupus.^7^ We highlight a case of a young lady with SLE who presented with worsening shortness of breath and a one‐month history of high‐grade fever with productive cough. After a series of investigations, bronchoscopic biopsy showed an endobronchial carcinoid tumor obstructing left upper lobe bronchus resulting in recurrent infective exacerbations from focal bronchiectasis.

## 2 CASE DESCRIPTION

A 30‐year‐old married woman with two children presented with one‐month history of intermittent fever with a maximum temperature of 102°F, associated with left‐sided sharp chest pain, which worsened with deep breathing and coughing. She had a productive cough with expectoration of whitish colored sputum and experienced shortness of breath while lying down in supine position. She also had a weight loss of four kilograms over preceding two month before current hospitalization. On systemic inquiry, she emphasized on moderate joint pain without rashes, photosensitivity or Raynaud's phenomenon. Of note, her obstetric history was significant for first‐trimester abortions. She also recieved treatment for Pulmonary Tuberculosis in 2014 and a repaired atrial septal defect (ASD) in childhood.

On examination, she was a young woman of thin‐built, who was unable to recline. Her vital signs showed a heart rate of 104 beats per minute, blood pressure 132/82 mm Hg, Temperature of 37.5 C, and respiratory rate of 24 breaths per minute on four liters of supplemental oxygen via nasal cannula. She had marked pallor, pedal edema and non‐scarring alopecia. She had decreased lung expoansion and breath sounds upto mid‐chest bilaterally, with coarse inspiratory crackles in the left upper chest. Her abdomen appeared distended without organomegaly but shifting dullness was present on percussion. Bowel sounds were audible. The remaining systemic examination was unremarkable.

## 3 INVESTIGATIONS

**FIGURE 1:**
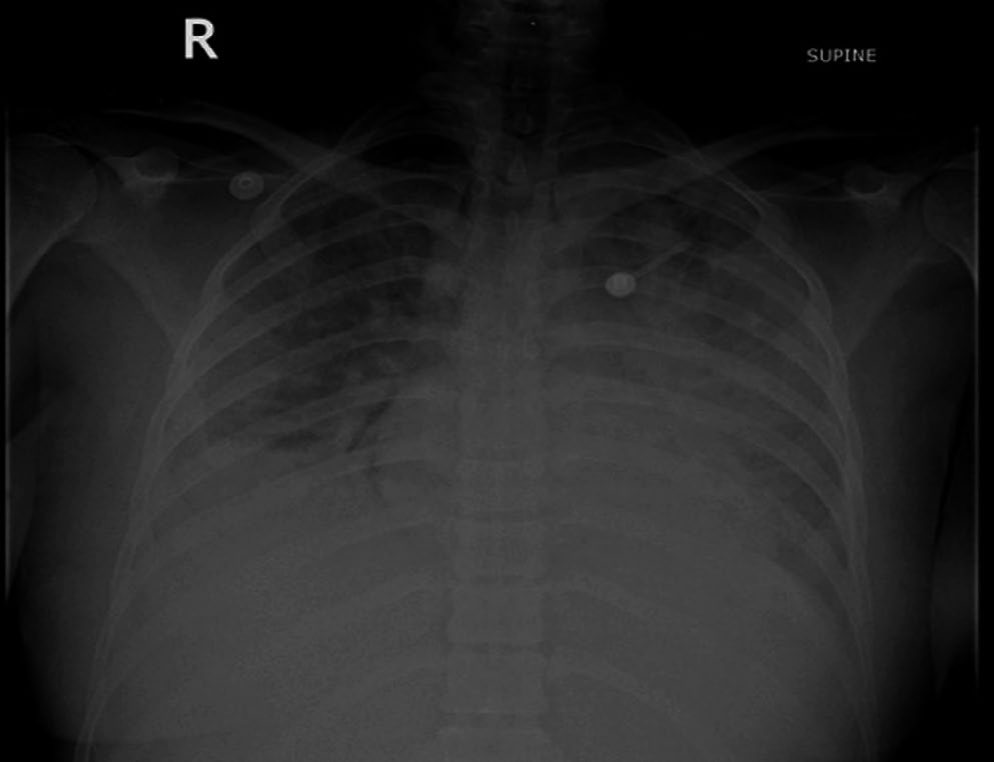
Chest X‐ray showing bilateral pleural effusion and dense infiltrate in the left lung

**FIGURE 2:**
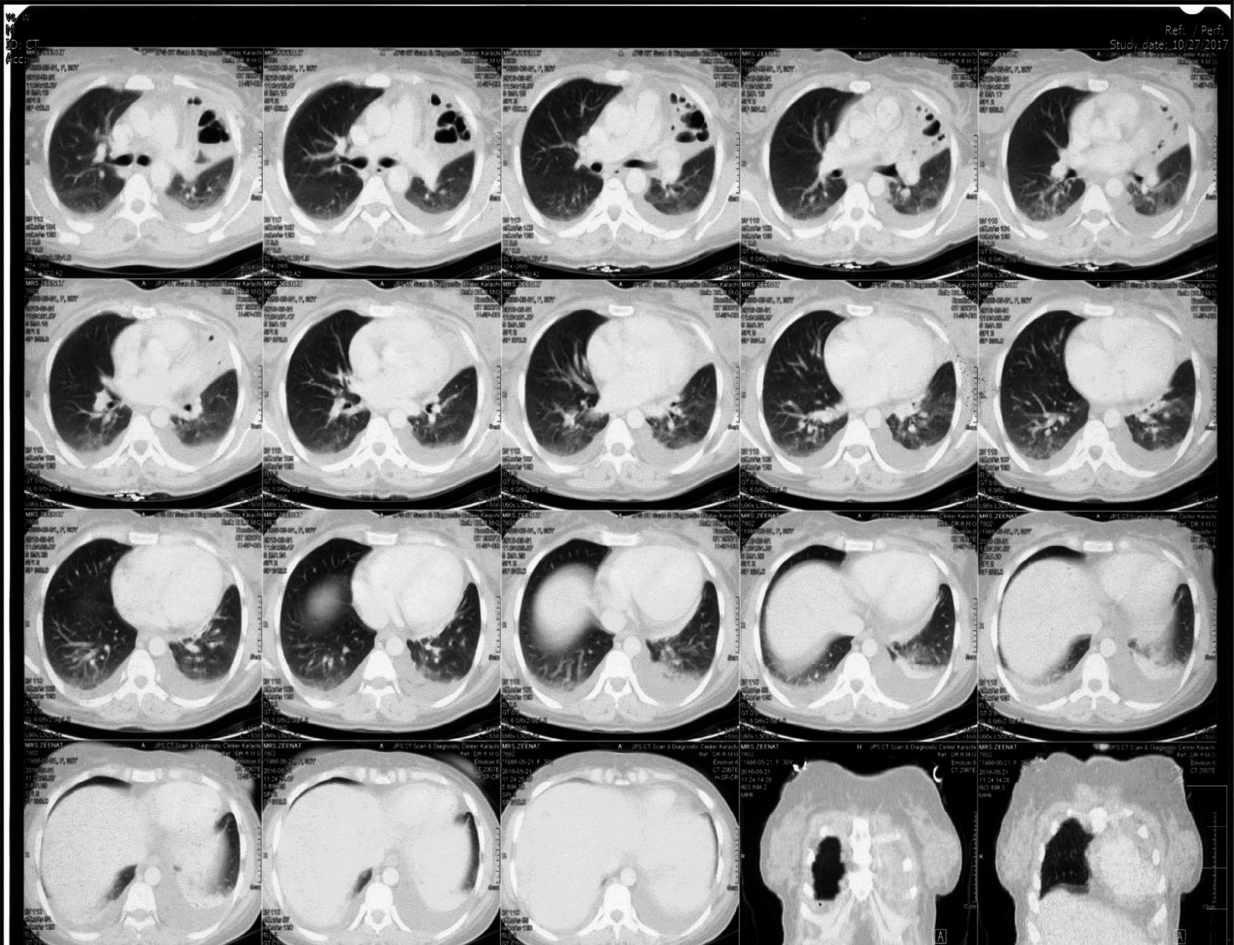
CT scan of chest with contrast redemonstrates bilateral pleural effusions and left upper lobe collapse with bronchiectasis

**FIGURE 3:**
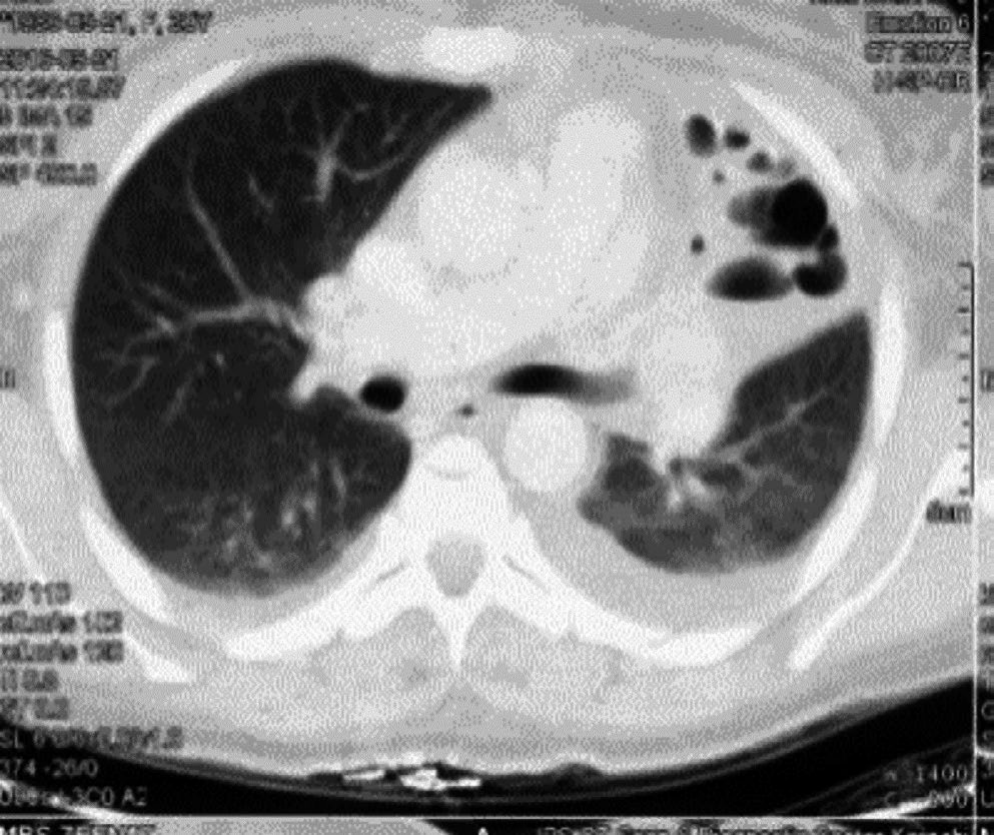
Left upper lobe bronchiectasis

**FIGURE 4:**
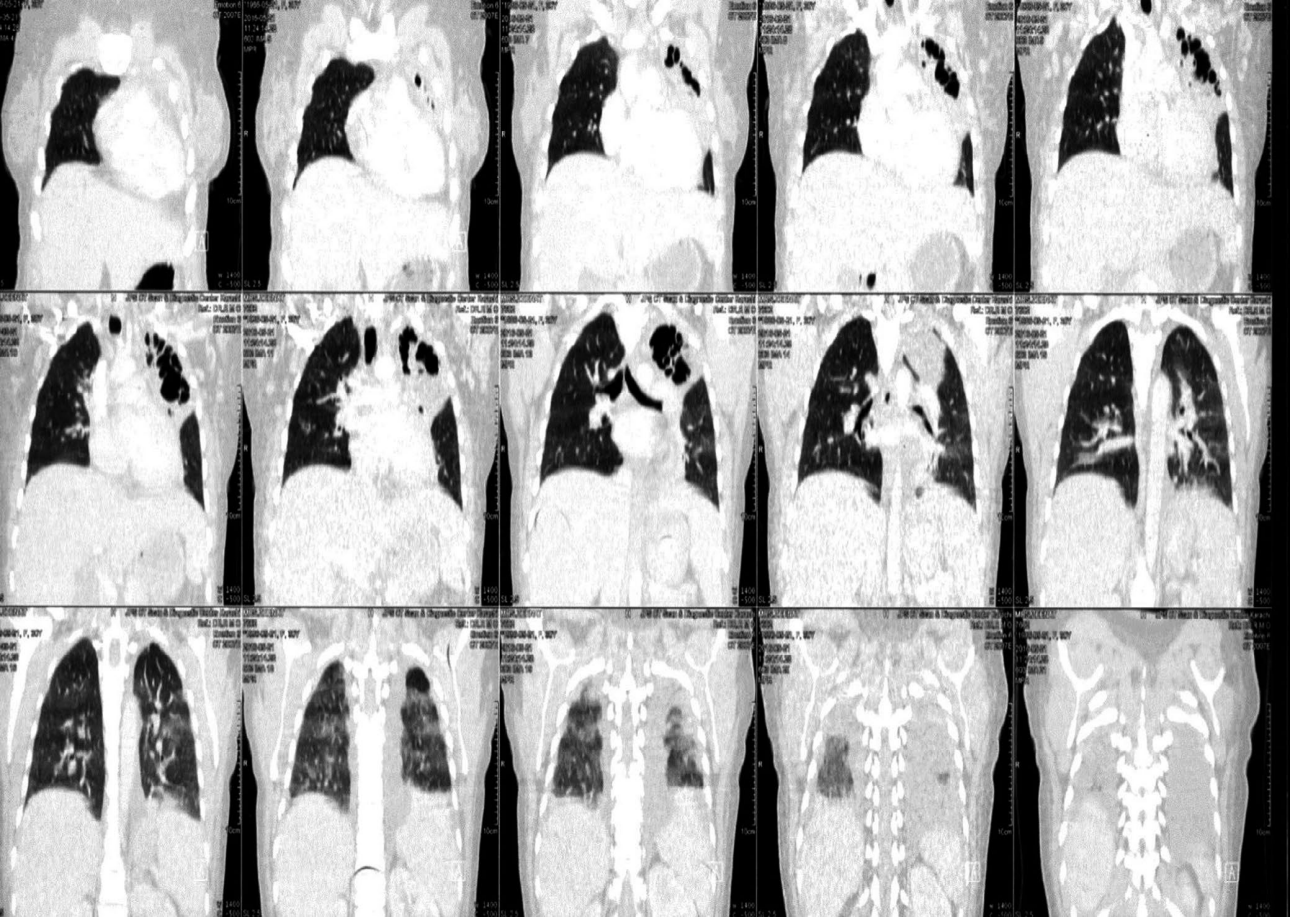
Left upper lobe bronchiectasis with underlying pleural effusions of lupus serositis

Her laboratory work‐up showed an elevated ESR(Table 1). There were bilateral pleural effusions on the chest radiograph (Figure 1). Her autoimmune profile showed a homogenous triple‐positive antinuclear antibody (ANA), elevated anti dsDNA titres and low level of complements C3 and C4.(Table 1). Diagnostic thoracentesis yielded 550ml clear yellow pleural fluid with an exudative picture (Table 2). A 12‐lead ECG showed sinus tachycardia and a transthoracic echocardiogram showed a trace circumferential pericardial effusion and preserved left ventricular systolic and diastolic function. We decided to seek clarity on her pulmonary manifestations and proceeded for a contract‐enhanced CT scan of the chest which showed brochiectasis in the upper and apical segments of the left lung and redemonstration of moderate pleural effusions bilterally (Figures 2,3 and 4).

**TABLE 1:** Investigations

Investigations	
Laboratory test	Result
Hb/Hct/MCV	11.2 g/dl/Hct 33.7/MCV 73.6fl
WBC (*N*%, L%, others %)	9,000/ul (*N* = 62.8%, L = 21.4%, M 10.2%)
Platelet count	266,000/ul
BUN	09 mg/dl
Creatinine	0.8 mg/dl
Electrolytes	Na +:135 meq/L K+: 4.9 meq/L Cl^−^:109 meq/L HCO3^−^:20
Urinalysis	WBCs 6/hpf, RBCs 10/hpf, protein+1
ALT	17 U/L
CRP	5.92 mg/dl
ESR	48 mm/hr
ABG	7.47/19.4/100/14.3/‐−7.6/95.7%
ANA	3+ Homogenous
Anti‐dsDNA	27.4 U/ml
Complements C3 and C4	0.27, 0.07 G/L
Anti‐Ro antibody	More than 100 U/ml

**TABLE 2:** Pleural fluid analysis

Pleural fluid analysis: Clear yellow 550 ml aspirated PF protein: 2,900 g/L PF LDH: 417 IU/L Serum protein: 5,400 g/L Serum LDH: 319 IU/L Cell counts: WBC: 400/cumm, Neutrophils: 20%, lymphocytes: 80%
Pleural fluid cultures: Negative
Pleural fluid AFB and GeneXpert: Negative AFB [acid‐fast bacilli]

**FIGURE 5:**
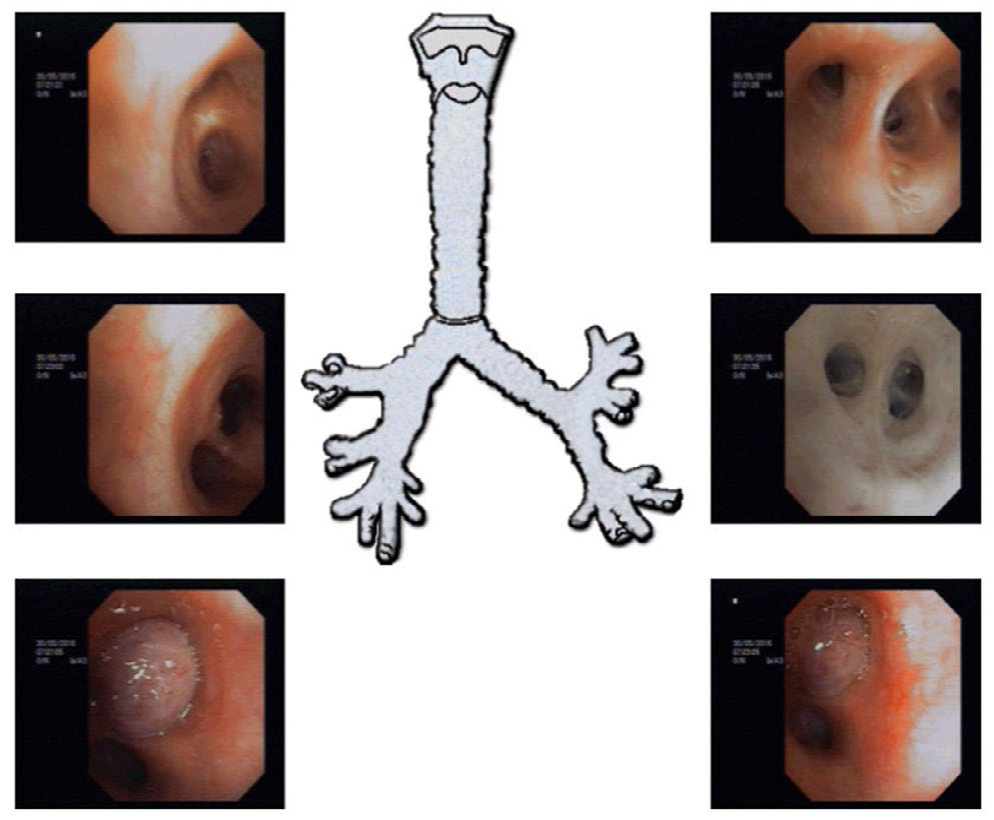
Fiberoptic bronchoscopy showing a well‐defined, shiny, rounded lesion in the left upper bronchus causing complete obstruction, bronchoscope could not pass beyond the lesion

**FIGURE 6:**
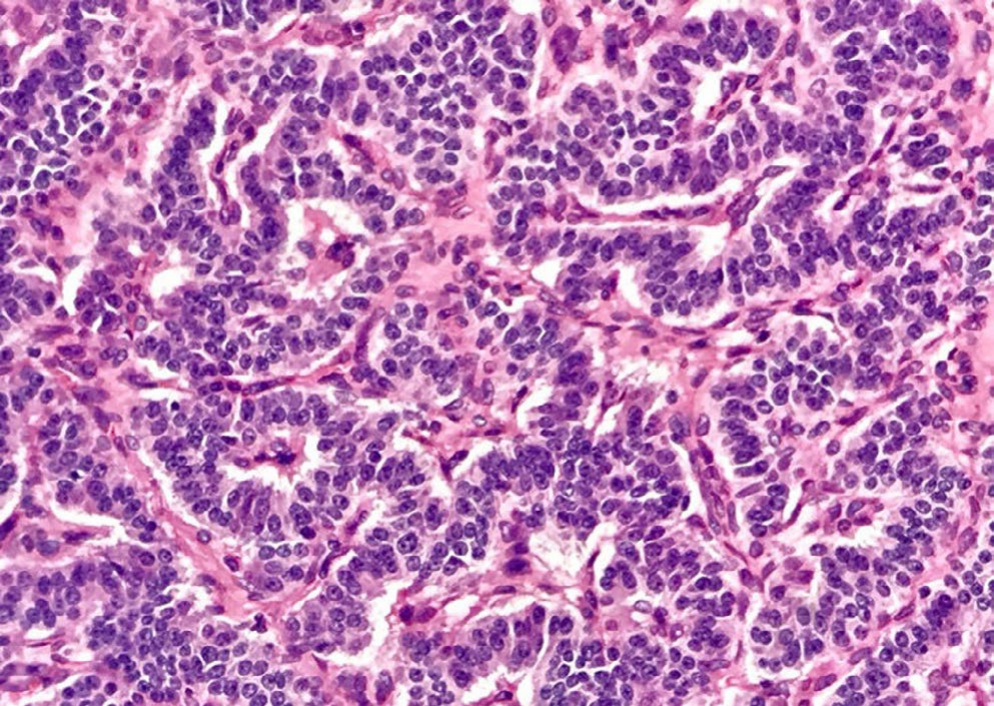
Photomicrograph of carcinoid tumor of the lung showing a characteristic growth pattern of neuroendocrine tumor; H &E: Histopathology shows sheets of cohesive cells with scant granular cytoplasm and oval‐stippled nuclei

**FIGURE 7:**
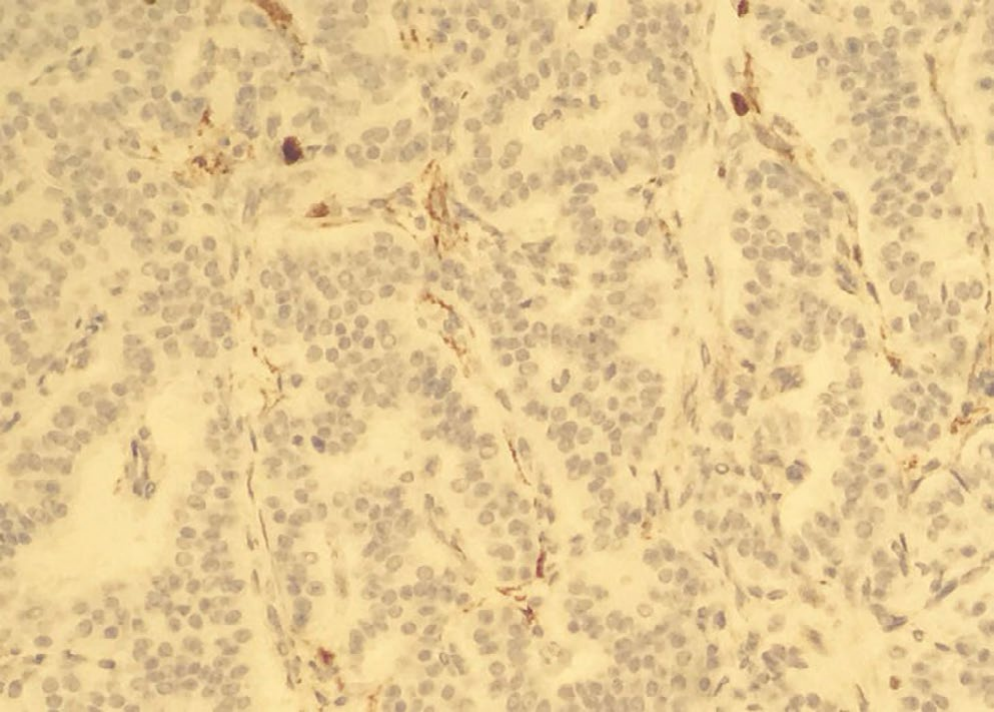
Abundant oxyphilic tumor cell cytoplasm

**FIGURE 8:**
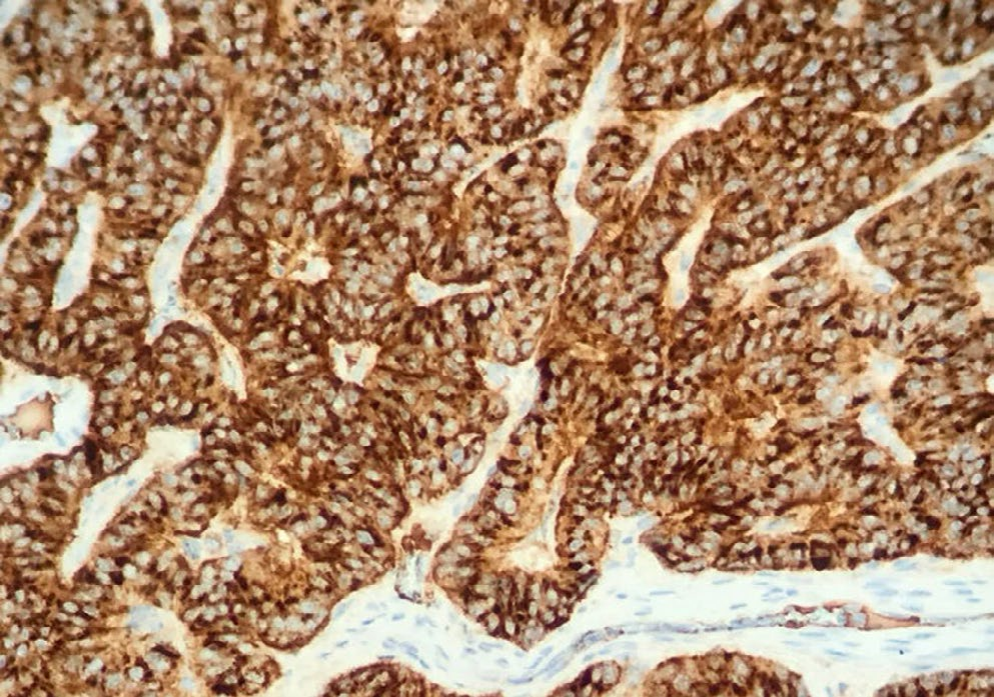
Positive staining for chromogranin A and synaptophysin

Fibreoptic bronchoscopy performed in succession showed a well‐defined,shiny, rounded lesion in the left upper bronchus resulting in complete luminal obstruction (Figure 5). The bronchoscope could not be advanced beyond this lesion, and a biopsy specimen was obtained. Histopathology showed an abundant oxyphilic tumor‐cell cytoplasm, positively staining for Chromogranin A and Synaptophysin (Figures 6,7 and 8). These characteristic findings confirmed the presence of an endobronchial carcinoid tumor.

## 4 DIFFERENTIAL DIAGNOSES

Our differential diagnoses included post‐tubercular bronchiectasis or a reactivation of pulmonary tuberculosis. Necrotizing cavitory pneumonia was the next differential in view of her clinical presentation with high grade fever and productive cough. Pulmonary manifestation of SLE was also a consideration given evidence of serositis and parenchymal lung involvement. Finally, the diagnosis turned out to be post‐obstructive focal bronchiectasis secondary to an endobronchial carcinoid tumor with lupus serositis.

## 5 TREATMENT

The endobronchial tumor was surgically resected by thoracotomy and left upper lobectomy. Postoperative pain was effectively managed with Naproxen and Tramadol infusion. She was also commenced on prednisone at a dose of 0.5 mg per kg per day, Hydroxychloroquine 200 mg twice a day, co‐trimoxazole DS tablet every other day as well as calcium and vitamin D3 supplementation. Prophylactic influenza and pneumococcal vaccinations were also administered to the patient following tumor resection.

## 6 OUTCOME AND FOLLOW‐UP

On follow‐up visits in the clinic, no tumor recurrence was found in serial CT scan at yearly follow‐up imaging. Her lupus disease activity is well‐controlled on stable doses of prednisone at 5mg/day, hydroxychloroquine 200 mg twice a day and Azathioprine 50 mg once a day.

## 7 DISCUSSION

Systemic lupus erythematosus (SLE) is a chronic systemic inflammatory autoimmune disease that can potentially affect many organ systems. Lungs can be involved in up to two thirds of patients with SLE at some point in their disease course.^8^ The most common pulmonary manifestation in SLE is pleural effusion. It occurs in 50% of the patients and is typically associated with pericardial effusion.^9^ A meta‐analysis shows that late‐onset SLE has more pulmonary manifestations such as serositis and interstitial lung disease than young peers with early‐onset SLE.^10^ The case we presented had a similar finding and the initial clinical impression was that it was a pulmonary manifestation (pleural effusion) of SLE with active disease. Her chest CT scan showed bronchiectatic changes that mimicked post‐tuberculosis bronchiectasis as tuberculosis causes structural alterations, such as scar formation, bronchial stenosis, and bronchiectasis.^11^

SLE is associated with a high risk of secondary malignancies.^7^ Several cohort studies report an increased risk of non‐hematologic malignancies encompassing lung, liver, head and neck, thyroid, vaginal/vulvar, cervical (cancerous, precancerous), dermatologic, bladder, renal, anal and pancreatic tumors.^7^ A higher risk of non‐Hodgkin's lymphoma is lupus is well known. Kotera et al. ^12^ reported a patient with gastric mucosa‐associated lymphoid tissue (MALT) lymphoma associated with a number of autoimmune conditions namely autoimmune gastritis, autoimmune thyroiditis, autoimmune hemolytic anemia, with underlying SLE.

Regarding lung malignancies in SLE, several studies demonstrate a high risk for lung cancer in SLE.^13^ A multicenter international cohort study of 9,547 patients with SLE found that the most common histologic type was adenocarcinoma (26.7%), followed by small‐cell carcinoma and squamous cell carcinoma with one case each of large‐cell carcinoma and carcinoid tumor.^13^

Neuroendocrine tumors account for only 0.5% of all malignancies, and carcinoid tumors are a subset of neuroendocrine tumors.^5^ The main primary sites are the gastrointestinal tract (62–67%) and the lung (22–27%).^14^ Ichiki et al. reported eleven cases of carcinoid tumors over 15 years of the patients undergoing surgical treatment for non–small‐cell lung cancer.^15^ Six patients had typical, and five had atypical carcinoid tumors.^16^

Several cases have been reported about gastric carcinoid tumors in association with SLE. A case report has highlighted a rare case of gastric carcinoid in a relatively young woman with autoimmune atrophic gastritis and systemic lupus erythematosus (SLE), treated definitively by endoscopic submucosal dissection (ESD).^16^ Papadimitraki et al. reported a case of multiple gastric carcinoids in a 23‐year‐old woman with systemic lupus erythematosus and atrophic autoimmune gastritis.^17^ Song et al. reported a similar case of a young 42‐year‐old woman with recurrent type I gastric carcinoid tumors who suffered from systemic lupus erythematosus.^18^ Interestingly, another report by Bhambani et al. described a unique patient with pituitary carcinoid in a patient with systemic lupus.^18^

Similarly, the case that we have described in our report demonstrates an atypical presentation of endobronchial carcinoid tumor in a young woman with SLE. To the best of our knowledge, no such case has ever been cited in the literature. A mechanism that raises the probability of carcinoid tumors in patients with underlying autoimmune disease is likely but warrants validation in a well‐designed prospective study.

## ACKNOWLEDGEMENT

I would like to acknowledge Dr Syed Faisal Mahmood, Consultant Adult infectious diseases at Aga Khan University for his kind referral and valuable contribution in excluding an infectious etiology in this patient.

## CONFLICTS OF INTEREST

There is no any conflict of interest of author or any of co‐authors.

## AUTHOR CONTRIBUTIONS

We affirm that all individuals listed as authors agree that they have met the criteria for authorship and agree to the conclusions of the study. NN managed the patient as a primary care physician and critically reviewed and revised the manuscript before submission. SA wrote the initial draft of the case report and performed literature search. TS was involved in the management of patient as a pulmonologist and reviewed the manuscript for content and clarity. SF was involved in the patient management as cardiothoracic surgeon and contributed to the content of the manuscript.

## ETHICAL APPROVAL

Informed consent was obtained from the patient regarding the report of her clinical scenario.

## CONSENT

We confirm that the patient consent has been signed and collected in accordance with the journal's patient consent policy. We will retain the consent form and will provide it if requested.

## Data Availability

Data sharing is not applicable to this article as no datasets were generated.
